# Concussions in Portuguese Professional Football: A Preliminary Epidemiological Study

**DOI:** 10.3390/diseases13100332

**Published:** 2025-10-08

**Authors:** André Moreira, Filipe Froes, Gonçalo Vaz, Alexandre Fernandes, Basil Ribeiro, Frank Mederos, Gabriel Nogueira, Hugo Almeida, Pedro Caetano, Pedro Prata, Ana Teixeira, Reinaldo Teixeira

**Affiliations:** 1 Health Department, Liga Portugal, 4250-540 Porto, Portugal; 2 Liga Portugal Concussion Working Group, 4250-540 Porto, Portugal; 3 Liga Portugal, 4250-540 Porto, Portugal

**Keywords:** concussion, traumatic brain injury, football, epidemiology, player safety

## Abstract

Introduction: Concussions are a growing concern in professional football due to their potential short- and long-term neurological consequences. Despite increasing global awareness, data on the epidemiology and clinical management of concussions in Portuguese football remain scarce. This preliminary exploratory study aimed to characterize the incidence, mechanisms, symptomatology, and medical response to concussions in Portugal’s Professional Football Leagues during the 2023/2024 season, based on reported cases. Methods: A retrospective observational analysis was conducted on head injuries reported by club medical teams during official matches in Liga Portugal First and Second Leagues. Collected variables included player position, time of injury, mechanism, symptoms, medical interventions and hospital referral. Results: Only six concussions were reported during official matches, with an overall incidence of 0.60 per 1000 player-hours. Most occurred in defenders, primarily due to head-to-head collisions, followed by ball impact, falls, and maxillofacial trauma. Injuries were more frequent during the final third of matches. Common symptoms included loss of consciousness, headache, and amnesia. Half of the players were referred to hospital care and underwent cranial CT scans. Among all variables analyzed, a statistically significant association was found between mechanism of injury and occurrence of amnesia (*p* = 0.014), with non-head-to-head impacts more frequently associated with amnesia. However, given the extremely limited sample size, this finding should be interpreted with extreme caution and requires replication in larger cohorts. Conclusions: This preliminary study suggests that defenders face a higher risk of head injuries, particularly from head-to-head impacts occurring late in matches. The prevalence of severe symptoms and the potential association between non-head-to-head impacts and amnesia highlight the need for more robust injury surveillance systems and underscore the importance of improved sideline assessment and return-to-play protocols. The findings emphasize the urgent need for comprehensive, standardized reporting mechanisms for concussions. Further research should explore long-term neurological effects and the effectiveness of preventive measures such as rule modifications, protective measures, and enhanced concussion management protocols, supported by more extensive and systematically collected data.

## 1. Introduction

Concussions have become a major concern in professional sports, particularly in high-contact disciplines such as football (soccer). A concussion, classified as a mild traumatic brain injury (TBI), occurs due to biomechanical forces transmitted to the head, resulting in a temporary disruption of brain function [1,2]. These injuries present immediate and short-term symptoms, including confusion, dizziness, and memory impairment, but can also contribute to long-term neurological consequences, such as cognitive decline, emotional instability, and an increased risk of neurodegenerative diseases like chronic traumatic encephalopathy (CTE) [2,3,4,5,6,7,8,9,10,11]. Despite being a globally celebrated sport, football exposes players to frequent head impacts, making concussions a significant risk at all competitive levels.

In professional football, headers, aerial duels, and accidental collisions are common, creating an environment where concussions frequently occur but often go unreported or mismanaged [12]. Cultural factors, such as the emphasis on toughness and perseverance, may discourage players from reporting symptoms, thereby increasing the risk of recurrent injuries and long-term damage [13]. Moreover, players may intentionally minimize or conceal symptoms out of fear of being removed from training sessions or match play, further complicating timely diagnosis and appropriate management [14,15]. These concerns underscore the need for rigorous epidemiological studies to better understand the incidence and management of concussions in professional football, where the intensity of competition and pressure to perform further elevate the risks [16].

Several international studies have analyzed concussion prevalence in football, with findings varying based on region, competition level, and study methodology. Pfister et al. estimated concussion incidence in professional football to range between 0.4 and 1.2 per 1000 player-hours, a significant figure considering the number of matches played annually [17]. Research from the English Premier League (EPL) has shown an increasing trend in concussion rates, with higher occurrences during matches compared to training sessions [18]. However, despite growing evidence, European football still lacks standardized concussion protocols, particularly regarding player education, assessment methods, and return-to-play guidelines, which are crucial for ensuring player safety [13].

In Portugal, football holds immense cultural and social importance, with thousands of professional players competing at the highest levels. However, despite the sport’s prominence, there is a lack of systematic research on the epidemiology of concussions in Portuguese professional football. While international studies provide valuable insights, country-specific data remains scarce, particularly in smaller footballing nations. This knowledge gap raises concerns regarding the adequacy of current injury management protocols and whether Portuguese clubs adhere to the latest international concussion guidelines, such as those proposed by FIFA and the International Concussion in Sport Group (ICSG) [1].

This study conducts a detailed epidemiological analysis of concussions in professional football in Portugal during the 2023/2024 season. The primary objective is to determine the incidence, risk factors, and outcomes associated with concussions among players in Portugal’s professional football Leagues. Analyzing concussion patterns in the Portuguese League is crucial for making evidence-based decisions that enhance player health and safety while minimizing long-term neurological consequences.

Given the increasing international research on sports-related concussions, this study has the potential to contribute valuable data to the global understanding of concussions in football. Ultimately, this study seeks to enhance player safety in professional football by providing an epidemiological foundation for improved concussion management. The data generated can support the development of targeted interventions that prioritize player welfare while maintaining the integrity and competitiveness of the sport.

## 2. Materials and Methods

This is a retrospective, observational study focusing on concussions sustained by active professional football players in Portugal’s First and Second Leagues official matches reported during the 2023/2024 season. These leagues collectively comprise 36 clubs (18 in the First League and 18 in the Second League), involving approximately 1000 registered athletes at the start of the season.

Liga Portugal prioritizes player welfare, and the head team physician of each club maintains exclusive clinical autonomy regarding player health decisions on the field. Referees are explicitly instructed to immediately interrupt play upon suspicion of a player sustaining a concussion, allowing the medical team sufficient time to conduct a thorough on-field assessment. This protocol is in full alignment with the international standards which mandates the immediate and permanent removal of any player displaying a visible sign of concussion to prevent the risk of further injury. The timeframe for a player’s return-to-play is not defined in the competition regulations but is determined as an individualized clinical decision by the head team physician. This decision is strictly guided by the latest FIFA Concussion Protocol and international standards, which require a structured, symptom-limited graduated return to play progression and final medical clearance.

Data were collected from cases voluntarily reported by club medical teams following head injuries sustained during official League matches, with medical staff being instructed to complete a standardized electronic form. This form was designed to capture detailed and specific information about each concussion, ensuring uniformity and reliability in reporting. The following data were collected for each concussion event: competition (First or Second League), player’s position on the field, minute of occurrence, mechanism of injury (e.g., head-to-head contact, fall, object impact), signs and symptoms (e.g., dizziness, confusion, loss of consciousness), removal from match (whether the player was taken out of the game), immobilization (use of cervical collar or other immobilization techniques), evacuation to hospital (if the player was transported to a hospital) and diagnostic imaging prescribed at hospital.

Head injuries analyzed in this study were those sustained during official Liga Portugal first and second league matches, where medical staff identified concussion-related symptoms and players underwent medical evaluation either during or after the match. Injuries were excluded if they occurred outside official matches or if they were not suspected to be concussion related.

Conducted in accordance with the Declaration of Helsinki, this study relied on anonymized and voluntarily reported data. Strict confidentiality measures were applied, ensuring that no individual player or club was identifiable in the analysis.

A descriptive statistical approach was applied to analyze incidence rates, injury mechanisms, positional risk factors, and clinical outcomes. The concussion incidence rate was calculated as the number of concussions per 1000 player-hours [7]. Continuous variables (e.g., time of injury) were presented as mean ± standard deviation (SD), while categorical variables (e.g., injury mechanism, symptoms) were expressed as absolute and relative frequencies (%). Comparisons between groups (e.g., positional differences, match timing) were analyzed using chi-square tests for categorical variables and *t*-tests or Mann–Whitney U tests for continuous variables, depending on data distribution. Statistical significance was set at *p* < 0.05. All analyses were conducted using SPSS Statistics Version 31.0 (IBM Corp., Armonk, NY, USA).

## 3. Results

### 3.1. Incidence of Concussions

During the 2023/2024 season of Liga Portugal first and second leagues, a total of only six concussion cases were reported by club medical teams, underscoring the preliminary nature of these findings. The overall concussion incidence rate, calculated as the number of concussions per 1000 player-hours, was 0.60. When analyzed separately, the incidence rate was 0.60 per 1000 player-hours in both Liga Portugal first and second leagues.

### 3.2. Player Position and Concussion Occurrence

Defensive players accounted for 83.3% of all concussion cases (5 out of 6), while the remaining 16.7% occurred in attacking players (1 out of 6). No concussions were reported among midfielders. These proportions, while suggestive, must be interpreted with extreme caution given the very small total number of reported cases.

### 3.3. Mechanism of Injury

The most common injury mechanism was head-to-head collision (50%), including impacts with opponents and teammates. The remaining cases resulted from impact of the ball (17%), head impact against the ground (17%), and maxillofacial trauma due to a kick to the upper jaw (17%).

### 3.4. Minute of Occurrence

The mean time of concussion occurrence was the 75th minute, with injuries recorded between the 40th and 91st minutes. The majority (67%) of cases occurred after the 75th minute.

### 3.5. Clinical Symptoms and Medical Response

The most frequently reported symptoms were loss of consciousness (67%), headache (67%), and amnesia (33%). One player (17%) experienced disorientation/confusion. Following medical assessment, 50% of the concussed players were removed from play and referred to the hospital for further evaluation, where cranial CT scans were performed in these cases. Among these, one player also underwent a cervical CT scan. The remaining 50% were also removed from the field of play and monitored by the team medical staff but did not require hospital referral.

### 3.6. Relationship Between Competition Level and Symptom Severity

The analysis examined whether there was an association between the competition level (First League vs. Second League) and symptom severity. Cases were categorized as severe (loss of consciousness + amnesia) or mild (headache + disorientation + visual disturbances). Among the three concussions recorded in First League, all (100%) were classified as severe, while in Second League, two cases (67%) were severe, and one (33%) was mild. Statistical analysis (Chi-square test, *p* = 0.500) showed no significant association between competition level and symptom severity (Table 1). However, it is crucial to note that the small sample size significantly limits the statistical power to detect true associations, if they exist.

### 3.7. Distribution of Concussions by Player Position

Regarding player position, defenders accounted for five (83%) of the six reported concussions, while the remaining case (17%) involved a forward. Symptom severity distribution within each position showed that four (80%) of the concussions among defenders were classified as severe, while the single case in a forward was also severe (Table 2). Statistical analysis (*p* = 0.833) indicated no significant association between player position and symptom severity. Nonetheless, it is imperative to acknowledge that the limited number of cases in this study substantially compromises the statistical power required to identify true associations.

### 3.8. Match Timing and Concussion Occurrence

Concussions occurred between the 40th and 91st minutes of play, with an average occurrence at the 75th minute. The majority (5 out of 6, or 83%) took place in the final third of the match, while only one (17%) happened during the middle phase (Table 3). Symptom severity did not significantly differ based on the moment of injury (*p* = 0.833), meaning there was no statistical association between match timing and concussion severity. Yet, the exploratory nature of this study, constrained by its reduced sample size, mandates extreme caution in interpreting these findings, as the absence of significance might reflect a limitation in power rather than a true absence of effect.

### 3.9. Mechanism of Injury and Hospital Referral

Injury mechanisms varied, with head-to-head collisions accounting for three cases (50%), while the remaining three resulted from head impacts against the ground (1 case), another player’s body (1 case), or the ball (1 case). Medical response data indicated that half of the players (3 out of 6) were evacuated to the hospital, where all underwent cranial CT scans, and one received a cervical CT scan (Table 4). However, statistical testing found no significant relationship between injury mechanism and hospital referral (*p* = 0.400). When grouping head-to-head collisions separately from other mechanisms, analysis still did not reveal a significant association with the need for hospitalization (*p* = 0.500). However, the diminutive N precludes the formulation of definitive conclusions regarding the presence or absence of certain associations, underscoring that these results should be considered indicative rather than conclusive.

### 3.10. Relationship Between Injury Mechanism and Symptom Severity

The most common mechanism among severe cases was head-to-head collisions (40%), while impacts against another player’s body, the ball, or the ground accounted for 60% (Table 5). Among concussed players experiencing amnesia, all cases resulted from mechanisms other than head-to-head collisions. This was the only variable where a statistically significant association was found (*p* = 0.014), suggesting that amnesia might be more associated with non-head-to-head impacts (Table 6). For other symptoms, including headache (*p* = 0.200), loss of consciousness (*p* = 0.500), disorientation (*p* = 0.233), and visual disturbances (*p* = 0.333), there was no statistical association between the injury mechanism and symptom occurrence. Nonetheless, given the extremely limited sample size, this findings must be interpreted with caution.

### 3.11. Player Position and Injury Mechanism

All cases of head-to-head collisions occurred among defenders, whereas 66.7% of concussions from other mechanisms involved defenders, and 33.3% involved forwards (Table 7). Although this suggests that defenders may be at increased risk of sustaining head-to-head collisions, statistical analysis did not confirm a significant association. Given the restricted sample, statistical inferences are limited, and the replication of these results in studies with greater statistical power is fundamental to confirm any observed trends.

## 4. Discussion

This study offers a preliminary epidemiological overview of concussion events in Portuguese professional football and contributes to the growing body of literature addressing sports-related concussion within elite male athletes. Crucially, the present findings are based on a severely limited sample of reported cases, which represents a significant methodological constraint. Therefore, while these preliminary data suggest important trends regarding injury mechanisms, player roles, match timing, clinical presentation, and medical response, they must be interpreted with caution. The primary value of this study lies in identifying initial patterns and highlighting critical areas for future, more robust research. Although the sample limitations, the findings reveal important trends regarding the injury mechanisms, player roles most affected, timing within the match, clinical presentation, and medical response.

The predominance of concussions among defenders (83%) aligns with existing literature indicating that defensive players are more frequently involved in aerial duels and high-contact scenarios, such as heading challenges and last-minute clearances [19,20]. This supports previous studies identifying defenders and defensive midfielders as high-risk due to their frequent involvement in aerial duels, high-speed challenges, and physically intense interactions. Some studies also identified defensive midfielders and goalkeepers as disproportionately affected, reinforcing the idea that tactical roles and on-field responsibilities directly influence exposure to concussive impacts [18,21].

The incidence rate observed in this study (0.60 per 1000 player-hours) is comparable to values reported in recent prospective studies in professional football [21]. Such similarities suggest a commonality in the biomechanical and tactical conditions that predispose players to concussions, despite possible differences in injury-reporting practices and medical infrastructure.

The low incidence rate observed in this preliminary study warrants prudence, as it could be an indicator of underreporting rather than a true reflection of concussion frequency. This interpretation is consistent with global literature, which documents the high prevalence of underreporting across male contact sports. This phenomenon is often driven by complex factors, including athlete culture, fear of professional exclusion, and a poor awareness of subtle symptoms. Furthermore, a methodological limitation is the exclusive reliance on official match reports. Considering that training exposure in professional football is typically four to five times greater than match exposure, the true overall concussion incidence could be underestimated.

Notably, some studies have pointed out the variability of concussion incidence across age groups, competition levels, and countries, often attributing the differences to underreporting and the lack of a standard definition or diagnostic approach [17]. Moreover, the analysis did not reveal statistically significant associations between competition level (First vs. Second League) and symptom severity, although First League cases were all classified as severe.

The analysis of injury mechanisms showed that head-to-head collision was the most frequent mechanism (50%). Importantly, these incidents involving defenders are not restricted to collisions with opposing players during general play; they primarily occur during aerial duels (e.g., set pieces and crosses), which includes the risk of teammate-to-teammate collisions while contesting the same ball. This supports previous findings where accidental head-to-head contact is a leading cause of concussions, often occurring during contested headers [17]. This nuance is crucial for developing targeted prevention strategies for set-piece scenarios.

Notably, our analysis identified a statistically significant association between non-head-to-head impacts (such as falls or collisions with the ground or ball) and the occurrence of amnesia (*p* = 0.014). This observation tentatively suggests that while these mechanisms may be perceived as “lower risk,” they can lead to significant neurophysiological effects [22]. However, it is imperative to reiterate that, due to the limited sample size and the retrospective nature of data collection, this finding should be considered exploratory. The current literature underlines the importance of not overlooking these mechanisms and explains how even low-threshold biomechanical forces can disrupt cerebral metabolic homeostasis, leading to significant clinical outcomes [4,23].

A noteworthy pattern was the timing of concussions, with the majority (83%) occurring in the final third of matches. This is likely related to increased fatigue, reduced neuromuscular control, and elevated competitive intensity late in the game, all of which may heighten susceptibility to injury [24,25]. This temporal pattern aligns with previous evidence that links physical and mental fatigue with an increased risk of injury [26]. Some authors highlighted the physiological decline in neuromuscular performance as matches progress, particularly in positions requiring constant high-intensity efforts [27]. The fatigue hypothesis is further supported by other studies, who observed similar clustering of injuries late in matches across multiple seasons [28].

Symptomatically, loss of consciousness and headache were most common, reported in 67% of cases, and 50% of all concussed players were referred to hospital settings for further evaluation. The consistent use of cranial CT scans highlights a cautious diagnostic approach. Half of the concussed players were referred to hospital settings for further evaluation. Despite international consensus statements, uniform implementation of concussion protocols remains a challenge [1].

A key point of this preliminary study concerns the absence of standardized data regarding the return-to-play duration. While the methodological section details that the decision is strictly an individualized clinical clearance guided by the latest FIFA and international protocols, there is no standardized registry of the recovery time or the specific progression for each case. Consequently, we were unable to perform an epidemiological analysis on the average recovery time or identify potential predictors of protracted recovery, highlighting a crucial area for future data collection improvement.

The literature is increasingly pointing to the long-term implications of sports-related concussion, including cognitive decline, mood disorders, and neurodegenerative diseases such as chronic traumatic encephalopathy. Moreover, one study has shown that athletes with previous concussions have a significantly increased risk of sustaining further musculoskeletal injuries, suggesting lingering neuromotor or cognitive deficits that compromise postural control or reaction time [29]. This risk is exacerbated by premature return-to-play decisions, particularly in the absence of objective recovery markers [30,31]. Despite these risks, return-to-play times in some settings have been reported as shorter than those recommended in international guidelines, which may reflect a more permissive or inconsistent clinical culture. This underscores the need for structured education and centralized concussion management systems [32,33].

Another critical point is the underuse of neurocognitive testing and baseline screening. While SCAT tools offer a structured framework for acute assessment, longer-term monitoring—especially of memory, attention, and processing speed—requires preseason baselines and post-injury comparisons. Incorporating such tools into club protocols could help better determine readiness for return-to-play and reduce recurrence risk [34,35,36,37,38].

While our retrospective data cannot evaluate individual susceptibility factors, the high prevalence of head-to-head collisions during aerial duels points toward the importance of considering fatigue of the neck musculature. Current research suggests that neck strength and timely muscle activation can be protective against concussion [39]. Future prospective studies should incorporate baseline neck strength assessments and explore the role of neck-specific training programs in mitigating concussion risk.

Beyond the acute incidents reported, this study underscores the necessity of considering the issue of repeated sub-concussive head impacts, particularly those resulting from routine heading of the ball. Although such impacts may not cause immediate symptoms, the cumulative effect is a growing concern in the literature due to its potential link to long-term neurocognitive impairment [40]. A comprehensive concussion protocol must therefore consider both acute symptomatic injuries and the cumulative biomechanical load associated with high-volume head impacts

Given the small sample size and the inherent limitations of reported retrospective data, these findings are exploratory and should be validated in larger, prospective datasets. The primary methodological limitation of this preliminary study is the low number of reported concussions, which inherently results in weak statistical power. Consequently, any observational findings related to the timing of injuries (e.g., occurrence in the second half), positional differences (e.g., defenders vs. forwards), or mechanisms of injury should be interpreted strictly as possible trends rather than conclusive statistical evidence.

Taken together, the findings strongly underscore the urgent need for improved concussion awareness, systematic sideline assessment protocols, and consistent medical decision-making regarding player removal and return to play. The current data powerfully reinforce calls for educational interventions targeting medical teams, coaches, and players alike to foster a culture that prioritizes neurological health over short-term performance. Finally, future research must urgently address the current severe gaps in injury reporting and monitoring.

Future research should move beyond retrospective analysis to adopt prospective, multi-season methodologies to achieve higher statistical power and capture the true incidence rate. Specifically, we endorse the need for studies that incorporate the use of impartial observers and objective measures to monitor collisions during both training and match play. This approach provides a more accurate, comprehensive assessment of concussion incidence, exposure, and recovery times in professional football.

The Consensus statement on injury definitions and data collection procedures in studies of football injuries provides a framework for consistent injury definitions and data collection in football, which would help generate more reliable epidemiological data [35]. The creation of a national registry of head injuries in professional football—aligned with FIFA, UEFA, and ICSG guidelines—would represent a fundamental step forward in both surveillance and player protection, mitigating the limitations observed in this preliminary study.

## 5. Conclusions

This study provides one of the first preliminary epidemiological insights into concussion events in Portuguese Professional Football Leagues, highlighting relevant patterns in injury mechanisms, player positions, symptom severity, and medical response. However, it is critical to emphasize that these findings are based on a limited sample of reported cases, which prevents any definitive conclusions or broad generalizations. Crucially, due to the low number of incidents, the findings should be viewed as preliminary trends. Nevertheless, the exploratory findings suggest that defenders are disproportionately affected, likely due to their frequent involvement in aerial challenges and direct physical contact. Head-to-head collisions appear to be the leading mechanism of injury, and the prevalence of severe symptoms—such as loss of consciousness and amnesia—underscores the clinical relevance of these events, even within this constrained dataset.

The observation that most concussions occurred in the final third of matches suggests a potential influence of fatigue and match intensity on concussion risk. These results reinforce the importance of implementing robust, evidence-based guidelines for the assessment and management of concussions in football.

Considering evidence suggesting an increased risk of subsequent injury following concussion, and the potential for long-term cognitive consequences such as chronic traumatic encephalopathy, this study highlights the importance of extending concussion management beyond the acute phase. It is crucial to adopt a culture of safety that includes player education, adherence to international guidelines, and long-term follow-up. Establishing an injury surveillance system based on standardized definitions and procedures will be essential for improving data reliability and facilitating international comparisons.

Future studies are urgently needed to expand the sample size significantly, incorporate video analysis, and assess long-term outcomes. In parallel, educational programs targeting players, coaches, and medical staff are essential to promote a culture of safety and ensure that concussion symptoms are consistently recognized, promptly reported, and systematically managed, both acutely and in long-term follow-up.

## Figures and Tables

**Table 1:** Relationship Between Competition Level and Symptom Severity.

			Symptom Severity	
			Mild	Severe
Competition	First League	Count	0	3
		% within Competição	0.0%	100.0%
	Second League	Count	1	2
		% within Competição	33.3%	66.7%
Total		Count	1	5
		% within Competição	16.7%	83.3%

**Table 2:** Distribution of Concussions by Player Position.

			Symptom Severity	
			Mild	Severe
Player Position	Defenders	Count	1	4
		% within Player Position	20.0%	80.0%
	Forward	Count	0	1
		% within Player Position	0.0%	100.0%
Total		Count	1	5
		% within Player Position	16.7%	83.3%

**Table 3:** Match Timing and Concussion Occurrence.

			Symptom Severity	
			Mild	Severe
Match Timing	Middle third of the match	Count	0	1
		% within Match Timing	0.0%	100.0%
	Final third of the match	Count	1	4
		% within Match Timing	20.0%	80.0%
Total		Count	1	5
		% within Match Timing	16.7%	83.3%

**Table 4:** Mechanism of Injury and Hospital Referral.

			Hospital Referral	
			No	Yes
Mechanism of Injury	Head-to-head collision with an opponent	Count	2	0
		% within Hospital Referral	66.7%	0.0%
	Head-to-head collision with a teammate	Count	0	1
		% within Hospital Referral	0.0%	33.3%
	Head impacts against another player’s body	Count	0	1
		% within Hospital Referral	0.0%	33.3%
	Head impacts against the ball	Count	0	1
		% within Hospital Referral	0.0%	33.3%
	Head impacts against the ground	Count	1	0
		% within Hospital Referral	33.3%	0.0%
Total		Count	3	3
		% within Hospital Referral	100.0%	100.0%

**Table 5:** Injury Mechanism and Symptom Severity.

			Symptom Severity	
			Mild	Severe
Mechanism of Injury	Head-to-head collision with an opponent	Count	1	1
		% within Symptom Severity	100.0%	20.0%
	Head-to-head collision with a teammate	Count	0	1
		% within Symptom Severity	0.0%	20.0%
	Head impacts against another player’s body	Count	0	1
		% within Symptom Severity	0.0%	20.0%
	Head impacts against the ball	Count	0	1
		% within Symptom Severity	0.0%	20.0%
	Head impacts against the ground	Count	0	1
		% within Symptom Severity	0.0%	20.0%
Total		Count	1	5
		% within Symptom Severity	16.7%	83.3%

**Table 6:** Chi-Square Tests.

	Value	df	Asymptotic Significance (2-Sided)	Exact Sig. (2-Sided)	Exact Sig. (1-Sided)	Point Probability
Pearson Chi-Square	6000	1	0.014	0.100	0.050	
Continuity Correction	2667	1	0.102			
Likelihood Ratio	8318	1	0.004	0.100	0.050	
Fisher’s Exact Test				0.100	0.050	
Linear-by-Linear Association	5000	1	0.025	0.100	0.050	0.050
N of Valid Cases	6					

**Table 7:** Association Between Player Position and Injury Mechanism.

		Head-to-Head (% Within Mechanism)	Other Head Impacts (% Within Mechanism)
Player position	Defenders	3 100.0%	2 66.7%
	Forwards	0	1 33.3%

## Data Availability

The data presented in this study are available on request from the corresponding author. The data are not publicly available due to privacy and ethical restrictions.
